# High-Content and High-Throughput Clonogenic Survival Assay Using Fluorescence Barcoding

**DOI:** 10.3390/cancers15194772

**Published:** 2023-09-28

**Authors:** Haibin Qian, Selami Baglamis, Fumei Redeker, Julia Raaijman, Ron A. Hoebe, Vivek M. Sheraton, Louis Vermeulen, Przemek M. Krawczyk

**Affiliations:** 1 Department of Medical Biology, Amsterdam University Medical Centers (Location AMC), Meibergdreef 9, 1105 AZ Amsterdam, The Netherlands; h.qian@amsterdamumc.nl (H.Q.); r.a.hoebe@amsterdamumc.nl (R.A.H.); 2 Cancer Center Amsterdam, 1081 HV Amsterdam, The Netherlands; s.baglamis@amsterdamumc.nl (S.B.); v.s.muniraj@amsterdamumc.nl (V.M.S.); l.vermeulen@amsterdamumc.nl (L.V.); 3 Laboratory for Experimental Oncology and Radiobiology, Center for Experimental and Molecular Medicine, Amsterdam UMC Location University of Amsterdam, Meibergdreef 9, 1105 AZ Amsterdam, The Netherlands; 4 Oncode Institute, 3521 AL Utrecht, The Netherlands; 5 Amsterdam Gastroenterology Endocrinology Metabolism, 1105 AZ Amsterdam, The Netherlands; 6 Institute for Advanced Study, University of Amsterdam, 1012 WX Amsterdam, The Netherlands

**Keywords:** clonogenic survival assay, high-throughput screening in 384-well plates, high-content screening

## Abstract

**Simple Summary:**

The Clonogenic Survival Assay (CSA) is pivotal in gauging cell survival and proliferative capacity in oncology research. However, it’s limited by its binary output and its time- and labor-intensive nature. We developed LeGO-CSA, a refined version of CSA, utilizing imaging of cell nuclei tagged with fluorescent markers which encode red, green, blue fluorescent proteins and their variations. This facilitates both high-content and high-throughput analysis, delivering a potent tool for cancer research, promising heightened accuracy and efficiency, with the potential to expedite drug discovery and offer deeper insights into cellular dynamics in cancer. This transformative approach can revolutionize cancer research methodologies, making them more efficient and insightful.

**Abstract:**

The Clonogenic Survival Assay (CSA) is a fundamental tool employed to assess cell survival and proliferative potential in cancer research. Despite its importance, CSA faces limitations, primarily its time- and labor-intensive nature and its binary output. To overcome these limitations and enhance CSA’s utility, several approaches have been developed, focusing on increasing the throughput. However, achieving both high-content and high-throughput analyses simultaneously has remained a challenge. In this paper, we introduce LeGO-CSA, an extension of the classical CSA that employs the imaging of cell nuclei barcoded with fluorescent lentiviral gene ontology markers, enabling both high-content and high-throughput analysis. To validate our approach, we contrasted it with results from a classical assay and conducted a proof-of-concept screen of small-molecule inhibitors targeting various pathways relevant to cancer treatment. Notably, our results indicate that the classical CSA may underestimate clonogenicity and unveil intriguing aspects of clonal cell growth. We demonstrate the potential of LeGO-CSA to offer a robust approach for assessing cell survival and proliferation with enhanced precision and throughput, with promising implications for accelerating drug discovery and contributing to a more comprehensive understanding of cellular behavior in cancer.

## 1. Introduction

Cancer remains a significant public health concern and a leading cause of death worldwide [1], despite advances in diagnostic techniques and therapeutic interventions [1,2,3]. As such, there is a growing need to deepen our understanding of the biological processes underlying oncogenesis and to translate them into novel strategies for cancer treatment and prevention.

The clonogenic survival assay (CSA), originally introduced and implemented by Puck and Marcus in 1956 [4], is a widely utilized technique in fundamental oncology research [5]. Its primary aim is to investigate the responses and proliferation of (cancer) cells under both controlled and treatment conditions. CSA relies on the counting of cell clones, ideally originating from single cells seeded earlier, to calculate the ability of these single cells to form progeny (i.e., clones) under different treatment conditions [5,6,7]. This approach provides a means to quantify the effectiveness of potential therapies and explore the mechanisms underlying cancer development and progression.

Despite the usefulness of the CSA in oncology research, it has several limitations, including the significant amount of time required for manual counting and the potential for errors and experimenter bias [8]. To address these challenges, researchers have developed various variants of the CSA with improved clone analysis techniques. Recent advances in microscopy techniques and imaging algorithms have made it easier to obtain high-quality images and perform accurate automatic clone quantification. Several open-source applications, including OpenCFU [9], the NIST’s Integrated clone Enumerator (NICE) [10], an ImageJ macro program [11], the circular Hough image transform algorithm (CHiTA) [12], a quantum-inspired machine learning clone classifier [13] and a simple automatic counter setup in combination with a flatbed scanner [14], have been developed and widely used in the field. However, while these applications are effective at counting clones, they are generally not designed to extract any additional information that could be obtained from analyzing individual clones, and cells within them, in more detail.

**Figure 1:**
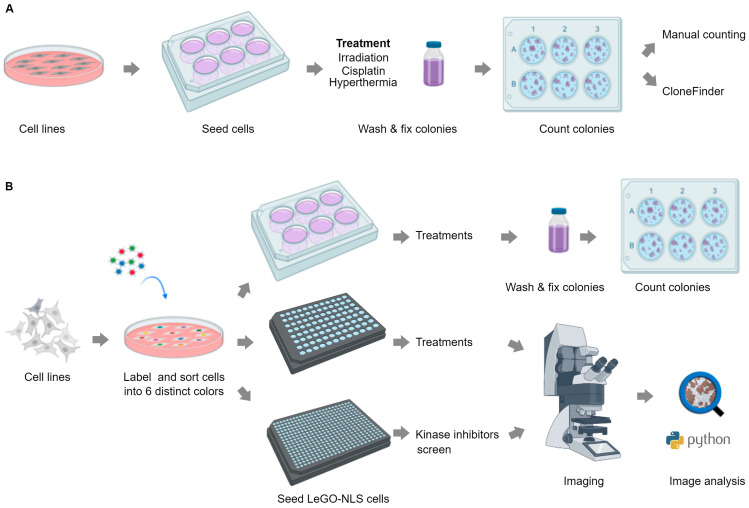
Schematic representation of experimental methodologies used in the study. (**A**) Cells in 6-well plates were fixed, stained and analyzed using CloneFinder 5.0 to extract clone and nuclei characteristics, and results were compared with manual counts. (**B**) Cells transduced with LeGO-NLS were seeded into both 6-well and 96/384-well plates. Manual counting was performed for the 6-well plates, while QuPath was used for clone and nuclei analysis in 96/384-well plates. Created with Biorender.com.

To enhance the scope and throughput of CSA, we first apply high-resolution analysis to classical CSA in 6-well-plate format using high-content microscopy, combined with a custom software tool named CloneFinder 5.0 (Figure 1A). This approach not only enables accurate, automated counting of clones, but also extraction of new characteristics for individual clones and nuclei such as size, compactness, roundness, distribution and DNA content. These features facilitate the investigation of distinct cellular behaviors within a single clone, as well as the properties of clones, providing novel insights into the mechanisms of clone behavior and survival.

In the second part, we extend this approach into an improved pipeline for a high-content and high-throughput clonogenic survival assay by integrating fluorescent cell barcoding and machine learning-based image segmentation [15,16] (Figure 1B). Using this pipeline, termed LeGO-CSA, we show that while providing additional information about individual cells and clones, reliable clone formation readout is feasible after only 3–4 days, significantly shortening the assay time. Moreover, we show that the format can be increased to 96- or even 384-well plates, enabling high-throughput analysis while maintaining the sensitivity and high content. As a proof of concept, we demonstrate the efficacy of LeGO-CSA in a small-scale, targeted 384-well screen, revealing subtle differences in the cellular effects of different kinase inhibitors.

## 2. Materials and Methods

### 2.1. Cell Culture

Four different human cancer cell lines were used in this study: SiHa and HeLa cervical cancer cell lines (ATCC, Manassas, VA, USA), RKO colorectal cancer cell line (Sanger Institute, Cambridge, UK) and Du145 prostate cancer cell line (ATCC). These cell lines were maintained in different growth media: EMEM (Lonza, Walkersville, MD, USA) for SiHA and HeLa, DMEM/F12 (Life Technologies, Waltham, MA, USA) for RKO and RPMI (Gibco, Waltham, MA, USA) for Du145. All media were supplemented with 10% fetal bovine serum (Gibco), 1% Penicillin-Streptomycin (Gibco) and L-glutamine (Gibco). The cells were incubated at 37 °C and 5% CO_2_.

### 2.2. Generation of Nuclear Derivatives of Lentiviral Gene Ontology (LeGO) Markers and Transduction

The nuclear localization signal (NLS) sequence from the human c-Myc proto-oncogene (3′ GGACGACGCTTCTCCCAGTTTAACCTG 5′) was cloned into LeGO-C2 (#27339, addgene, Watertown, MA, USA), LeGO-V2 (#27340, addgene) and LeGO-Cer2 (#27338, addgene) vectors, encoding mCherry, Venus and Cerulean maker genes, respectively, to enable nuclear visualization [17,18,19]. Standard DNA cloning protocols were employed for the cloning process. As previously described in published studies [15,20], lentiviral gene ontology (LeGO) markers were transduced into RKO and SiHA cells. Briefly, 50,000 cells were seeded per well in a 12-well plate with 1 mL of culture medium and incubated at 37 °C and 5.0% CO_2_ for approximately 24 h until they reached around 70% confluency. Then, 50 μL of concentrated lentivirus containing LeGO-NLS DNA was added to 1 mL of culture medium in the presence of 8 μg mL^−1^ polybrene (Sigma-Aldrich, St. Louis, MO, USA) and incubated overnight at 37 °C and 5.0% CO_2._ The lentivirus was generated using standard protocols [15].

### 2.3. Irradiation

All samples were subjected to irradiation with CellRad from Precision X-ray (Madison, CT, USA) at the specified doses.

### 2.4. Kinase Inhibitors Screen

To determine the optimal seed density for high-throughput studies, cells were cultured in a 96-well plate with a seeding density spanning from 100 to 350 cells per well, under an environment composed of 37 °C temperature and 5.0% CO_2_. Concurrently, 6-well plates were utilized to seed cells for CSA. After incubation overnight, treatments of cisplatin (Sigma-Aldrich) and 5-fluorouracil (5-FU, Sigma-Aldrich) were introduced. The cells were treated with cisplatin at a concentration of 3.3 μM, administered for a span of 3 h, whereas 5-FU was given at a concentration of 5 μM, imposed for an extended period of 72 h. Images from the 96-well plates were procured on the 2nd, 4th, 6th and 8th day post-seeding (considering the seeding day as Day 0).

A set of kinase inhibitors (a kind gift from Jan Paul Medema group), sourced from Selleck, are comprehensively listed in Table S1. This assortment includes the respective pathway details. After 24 h of incubation period with the inhibitors, the images from the 384-well plates were collected on the 4th day of seeding (again considering the seeding day as Day 0).

### 2.5. Imaging

Six well plates were examined using a Leica DMI8 microscope (Wetzlar, Germany), in phase-contrast mode at a 10× magnification for CloneFinder 5.0 analysis, using a standard DAPI filterset. Cells tagged with LeGO-NLS were imaged employing a Leica Thunder Wide Field Fluorescence Microscope also at 10× magnification using Quad Filter Block (CYR71010) for Channel 1 (Em: 642 nm, Exposure time: 100 ms), Channel 2 (Em: 539 nm, Exposure time: 100 ms) and Channel 3 (Em: 473 nm, Exposure time: 300 ms) for imaging mCherry, Venus and Cerulean, respectively.

### 2.6. Image Analysis

CloneFinder: Manual and automatic methods were employed for the analysis and quantification of six well-plate images. As for manual processing, cell clones were counted as per the following process: once the medium was removed, the wells were rinsed with PBS and stained with 1 mL crystal violet solution for thirty minutes. Subsequent to washing with distilled water, the wells were left to dry overnight. Any cluster containing 50 or more cells was considered to be a clone, and clones were manually counted using a microscope.

An automated high-throughput manner for counting cells was realized through the developed software, CloneFinder 5.0, using Matlab R2019a. Automation was enabled by configuring entire well-plate scans for the analysis. To guarantee precise data acquisition from the imaged well plate, several pivotal procedures were performed: recognizing and correcting differences in illumination caused by inconsistent camera sensitivity or inhomogeneous illumination over the well plate; setting an intensity threshold to differentiate cell nuclei from well edges and other image objects using the intensity derivative; ensuring a 10% overlap between frames to prevent data loss; integrating frames with matching data in the overlap regions to counter double counting of cross-border clones; and employing a watershed algorithm to separate nuclei in regions with high nuclei densities. In particular, due to the uneven illumination caused by the optics used, the background in the fluorescence images formed a gradient, with values decreasing toward the edges of each image. To normalize the background, we first determined its value at 20 locations in the image where there were no objects present (this was detected using an initial rough thresholding with Otsu method), and then we interpolated the background values in-between the measured points. The interpolated values were then subtracted from the value of each pixel.

LeGO-NLS: Cells were seeded in both 96-well plates (Greiner, Sensoplate glass bottom, Kremsmünster, Austria) and 384-well plates (Greiner, μClear) and imaged using a Leica THUNDER microscope. To ensure the sharpest images, adaptive focus control built into THUNDER was used for imaging the cells in the 96-well plates, while z-stacking was applied to the cells in the 384-well plates. Following image acquisition, custom software was utilized to preprocess and convert the acquired images into TIFF format. For the identification of nuclei, Qupath with StarDisk, a plug-in employing deep learning algorithms, was utilized (Supplementary File S1. Qupath image analysis).

Subsequently, the cells were manually categorized into seven classes, including six different color classes and one class for trash objects. To automatically classify cell-like objects into the predefined classes, a decision tree algorithm was employed with default settings. This non-parametric supervised learning method facilitated the training and classification of cells into their respective categories based on the provided classes. After exporting the digitized cell information, further analysis was conducted using the unsupervised algorithm known as Density-Based Spatial Clustering of Applications with Noise (DBSCAN). The main parameters employed in DBSCAN were the nuclei distance calculated from their location and the minimum sample numbers, which were manually determined and varied on different days.

Finally, unsupervised hierarchical clustering was applied to cluster different treatments into three classes based on the standardized results of both clone and nucleus characteristics.

### 2.7. Data Analysis

CloneFinder 5.0 software, as well as R and Python scripts for data analysis, will all be made available upon request.

### 2.8. Statistical Analysis

Statistical analyses were performed using GraphPad Prism 9. Significance was assessed using ANOVA, followed by a post-hoc test known as TukeyHD. The notation employed for reporting the results is as follows: “ns” denotes not significant, “*” signifies *p* < 0.05, “**” represents *p* < 0.01, “***” indicates *p* < 0.001 and “****” denotes *p* < 0.0001.

## 3. Results

### 3.1. Detecting and Analyzing Fluorescently-Stained Nuclei in Large-Format Images of Classical CSA Vessels

In order to establish methods for the high-throughput and high-content analysis of clonal growth in vitro, we first applied a brute-force approach: we performed a classical CSA in 6-well plate format [5], then stained cell nuclei with Hoechst 33342 and imaged entire wells using tile-scanning. To process and analyze these images, we developed a custom Matlab application that we term CloneFinder 5.0. Because of the generally low cell density in the plates, often resulting in low (or no) signal in the tile overlap areas, stitching the tiles using available standard software resulted in considerable tile misalignment. Instead, tiles were thus stitched together using a custom approach, whereby clones were first located using a low-resolution image consisting of tiles merged as-is (without alignment), and then only tiles containing clones were stitched together using the signal in overlapping regions. Following stitching, the background was normalized and cell nuclei were detected using the watershed algorithm. The images were then analyzed, yielding the exact location, surface area, total intensity and roundness of each cell nucleus. Nuclei were then assigned to clones, and parameters including clone compactness, surface area and the number of cells were determined for each clone.

**Figure 2:**
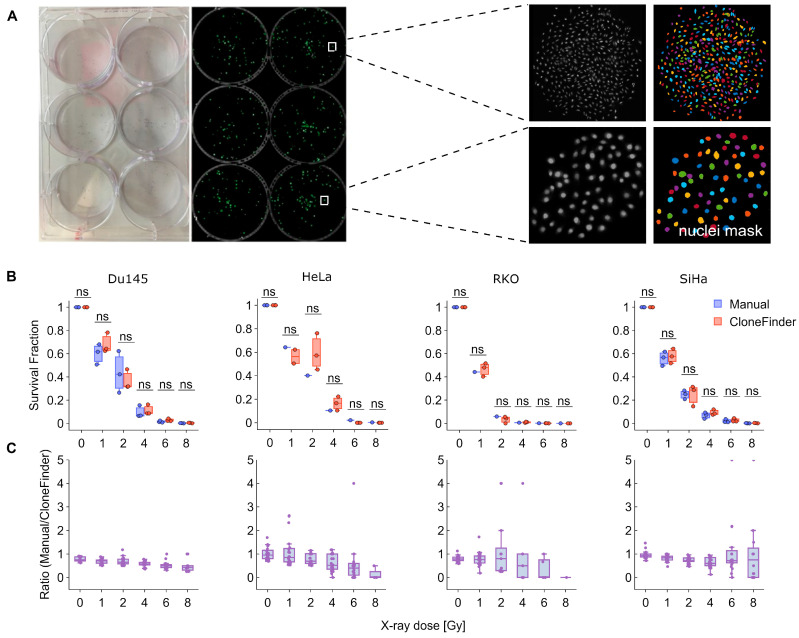
High-content analysis in classical CSA. (**A**) Left two images show the assay plates and the merged tilescans, scanned at 10× magnification. The right panel shows the raw scanned images of two clones and the masked images used to distinguish cell nuclei generated using CloneFinder 5.0. (**B**) Clonogenicity analysis after manual and automatic counting of clones for four different cell lines exposed to increasing doses of X-rays. N = 3. (**C**) Ratio of survival fractions from B. N > 3. ns: not significant.

To validate CloneFinder 5.0, we performed a classical CSA in 6-well plates, with cells exposed to 1–8 Gy of X-rays (Figure 2A). After approximately one week, all plates were analyzed by manually counting the clones that contained at least 50 cells. In parallel, the plates were fixed, stained using Hoechst 33342 and imaged in their entirety, after which the images were analyzed using CloneFinder 5.0. The plating efficiency and surviving fraction were then calculated based on data obtained from both manual counts and CloneFinder 5.0.

The results consistently show that the two methods produced similar clone counts in most of the cell lines studied, with no significant differences observed (Figure 2B). However, manual counting slightly (and not significantly) underestimated the number of clones, particularly at higher X-ray doses, where fewer clones were present (Figure 2C).

### 3.2. Detailed Analysis of Clone Characteristics Reveals That Classical CSA May Underestimate Clonogenicity

**Figure 3:**
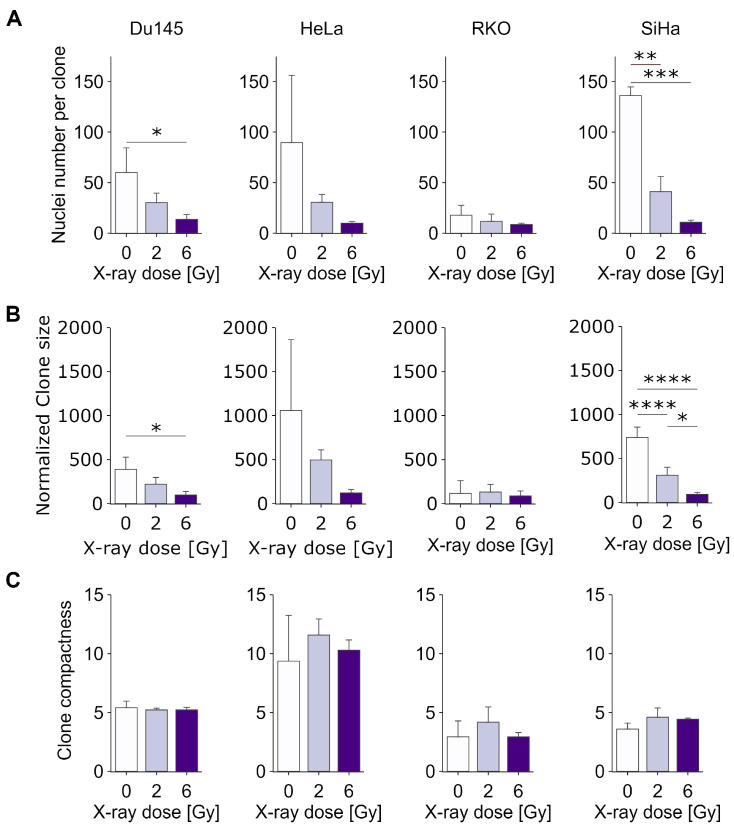
Changes in clone characteristics following exposure to increasing X-ray doses. (**A**) Average number of nuclei per clone; (**B**) average normalized clone area; (**C**) average clone compactness, representing clone area relative to cell number. N = 3. *: *p* < 0.05, **: *p* < 0.01, ***: *p* < 0.001, ****: *p* < 0.0001.

To deepen the analysis of clonogenic cell growth and survival in vitro, we then determined three additional parameters describing each individual clone: cell number per clone, clone area and clone compactness, representing the number of cells per normalized clone area. Values from all clones of each experimental condition (averaged per replicate) were used for the direct comparisons shown in Figure 3, while the detailed data distributions, showing all clones from all replicates, can be found in Supplementary Figure S1. The results show that the average number of cells per clone decreases considerably as the radiation dose increases (Figure 3A), with the decrease in RKO cells being comparatively smaller than in the other cell types. In all cases, we detected a relatively smooth distribution of clone sizes, ranging from a few cells to a few hundred cells, with large numbers of clones below the threshold of 50, which is generally (and arbitrarily) applied in classical CSA. This suggests that a significantly larger number of cells, beyond those detected using classical CSA, might possess the capability to form viable clones. This, in turn, suggests that the classical assay may considerably underestimate the clonogenic potential, which is especially important in the context of predicting cancer treatment efficacy in vitro. As expected, the clone size (Figure 3B) decreased proportionally to the number of cells per clone, resulting in generally unchanging clone compactness (Figure 3C), indicating that, on average, cell migratory and adhesion properties do not change significantly upon irradiation. Interestingly, however, there were considerable differences in the distributions of clone compactness between HeLa and other cell lines (Supplementary Figure S1), with HeLa showing a much broader peak, suggesting increased heterogeneity. This could reflect a larger clonal heterogeneity of HeLa, at least with respect to cell mobility and/or cell–cell or cell–surface adhesion.

### 3.3. Analyzing Properties of Individual Nuclei Reveals Changes in Cell Fate and Cell Cycle Status

In addition to clone-level features, our approach allows for the analysis of multiple parameters characterizing the individual nuclei, both in aggregate as well as in selected clones. Here we focus on three different parameters: total fluorescence intensity, area and roundness.

**Figure 4:**
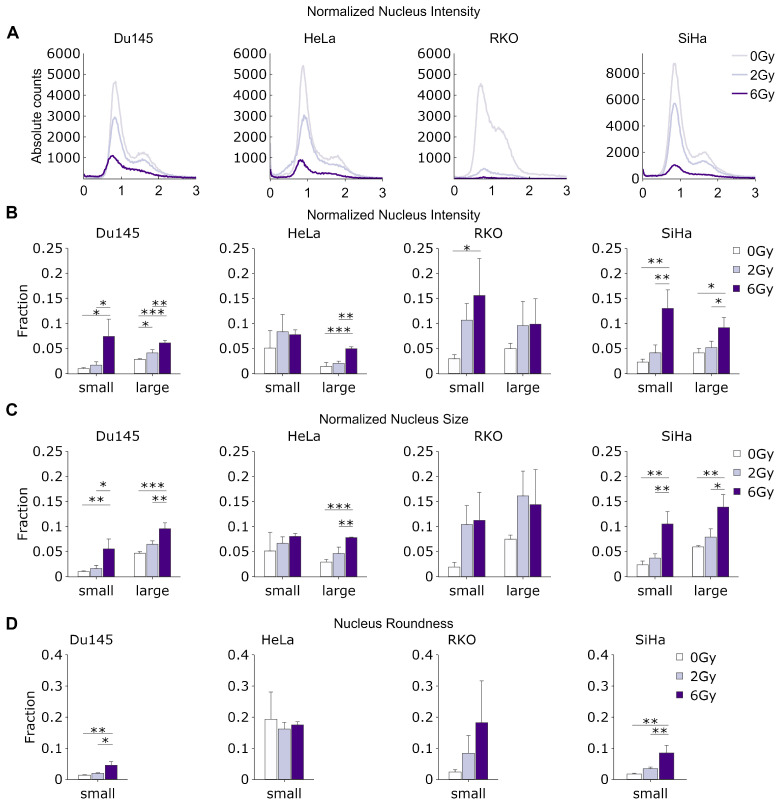
Analysis of individual nuclei in classical CSA reveals cell fate and cell cycle status. (**A**) Distributions of normalized total nuclear intensity, showing changes in DNA content and position in the cell cycle with increasing radiation dose; (**B**) changes in the relative fraction of small (<0.5) and large (3–10) nuclei, as determined by their median normalized total fluorescence intensity; (**C**) changes in the relative fraction of small (normalized area <0.5) and large (normalized area 2–4) nuclei; (**D**) changes in the relative fraction of nuclei with low (<0.5) roundness. N = 3. *: *p* < 0.05, **: *p* < 0.01, ***: *p* < 0.001.

As the intensity of Hoechst staining is proportional to the total amount of DNA in cell nuclei, by quantifying the total normalized fluorescence intensity, we were able to determine the total DNA content in each individual nucleus. This, in turn, enabled us to construct DNA content histograms, which provide information about the relative proportion of cells in the different phases of the cell cycle. In all cell lines, increasing the radiation dose led to the considerable flattening of the DNA content histograms, with a decreasing G1 fraction relative to the S/G2 fraction, likely caused by the increasing frequency of cells undergoing cell cycle arrest at the G1/S, S/G2 and G2/M checkpoints (Figure 4A).

We then focused on the changes in the relative fraction of low-intensity objects (smaller than 0.5 of normalized median nucleus intensity), which mostly represent small or fragmented nuclei, DNA fragments and micronuclei (Supplementary Figure S2) originating from unrepaired DNA damage. Interestingly, we observed a strong increase in the relative abundance of these small objects with an increasing radiation dose (Figure 4B).

We did not observe significant variations in either the mean or median cell size, although the SiHa and HeLa cells exhibited a slight trend toward a decreased median roundness and increased nucleus area with rising radiation doses (Supplementary Figure S3). The increase in nuclear size could potentially be attributed to the induction of senescence via higher radiation doses or alternative factors such as endo-replication, particularly post-DNA damage. Meanwhile, the decrease in roundness could signify nuclear fragmentation and deformation due to the presence of mis- or unrepaired DNA damage. Indeed, the relative frequency of abnormally large nuclei (larger than 200% of the median normalized area), which may represent senescent or quiescent cells—which often have large, flat nuclei—was highly and significantly increased in the irradiated cells (Figure 4C). Likewise, when we investigated the relative frequency of abnormally misshapen nuclei (roundness < 0.5), we found a considerable increase with higher radiation doses in all cell lines (Figure 4D).

In summary, the high-content analysis of clone and cell properties allows for the extraction of important parameters describing cell behavior and fate after exposure to ionizing radiation, in addition to clonogenic potential.

### 3.4. Clonal Growth Analysis Using Multicolor Fluorescent Barcoding

**Figure 5:**
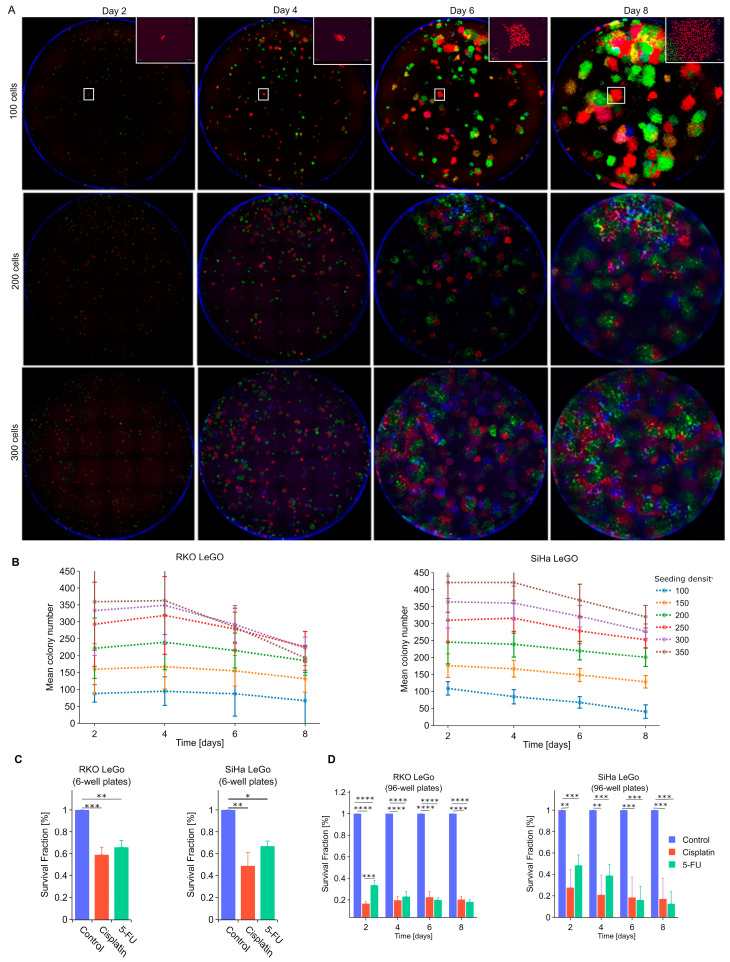
Tracking clonal growth using fluorescent cell barcoding. (**A**) Representative images of single wells from a 96-well plate at three seed densities (100, 200 or 300 cells per well) captured over time at 10× magnification, with a specific clone being selected and zoomed in on for detailed examination. (**B**) Quantification of changes in absolute clone numbers at various seed densities over time. (**C**) Survival fraction analysis based on manual clone counting in 6-well plates (classical CSA). (**D**) Survival fraction assessment at seeding density of 200 cells per well in 96-well plates over time. N = 3. *: *p* < 0.05, **: *p* < 0.01, ***: *p* < 0.001, ****: *p* < 0.0001.

The use of fluorescence microscopy for the high-content analysis of clone and cell characteristics allows for the identification of individual clones without necessitating a large number of cells per clone, in contrast to the traditional methods used for clonogenicity quantification [5]. We next relied on this characteristic to achieve two objectives: (1) allow for the analysis of smaller clones at earlier time points to shorten the assay time and (2) enable analysis in multi-well cell culture vessels for increased throughput, both while maintaining the high-content nature of the assay. A major challenge related to the second objective was that a smaller surface of multi-well vessels (i.e., 96- or especially 384-well plates) requires a much higher clone density relative to the 6-well plates used in our earlier, large-format analysis. This, in turn, leads to clones often touching and overlapping with each other, such that their separation is difficult. This can be partly compensated for if smaller clones are analyzed at earlier time points (objective 1) but is not efficient when larger numbers of clones are growing in small-area vessels. Inspired by the fluorescent lentiviral gene ontology (LeGO) cell barcoding system [21], we, therefore, generated SiHa and RKO cell lines stably expressing six different combinations of red (R), green (G) and blue (B) fluorescent proteins: R, G, B, RG, RB and GB. This involved individual transductions of cells with the six different combinations of LeGO constructs, followed by mixing at an equal ratio. The clone color served as an additional dimension along which we could distinguish individual clones in dense cultures (Figure 5).

To analyze clonogenic growth, cells were then seeded, at different densities, into glass-bottom 96-well plates and imaged in red, green and blue channels using a Leica Thunder microscope. The images were subsequently analyzed using QuPath, utilizing its built-in machine/deep learning algorithm to distinguish nuclei and classify them into different categories based on their fluorescent colors. To detect individual clones, nuclei locations were subjected to unsupervised clustering using the DBSCAN algorithm [22]. In parallel, for some experiments (Figure 5C), classical clonogenic assays in the 6-well format were performed using the same LeGO cell lines.

The optimal seeding density for the high-throughput LeGO-CSA was determined by imaging clones growing from between 100 and 350 cells/well (Figure 5A) and determining the clone number over time (Figure 5B). We found that for both cell lines (and most seeding densities), the mean clone number was comparable on days 2 and 4 and started to decrease on days 6 and 8, especially for initial densities above 200. As no treatments were applied in these initial experiments, the decrease in clone number was likely a consequence of clone merging and our inability to distinguish the individual clones; this indeed appeared to be the case after manual verification (not shown). We thus selected 200 cells/well as the optimal seeding density because this appears to be the highest density producing a stable clone number across all days.

To validate the LeGO-CSA, we compared its results to the results of classical CSA after treatment with cisplatin and 5-FU. As shown in Figure 5C,D, even though similar trends were observed using both assays, generally lower survival fractions in drug-treated samples were measured via LeGO-CSA. The difference could be potentially attributed to the different surfaces and coatings used in plastic 6-well plates and glass-bottom 96-well plates, which can considerably affect cell proliferation. However, as we were mostly interested in relative clonogenicity changes (compared with untreated controls), we did not pursue optimizations of cell culture vessels to decrease the spread between classical and LeGO-CSA.

### 3.5. LeGO-CSA Enables Multiparametric Kinetic Analysis of Clonal Growth

**Figure 6:**
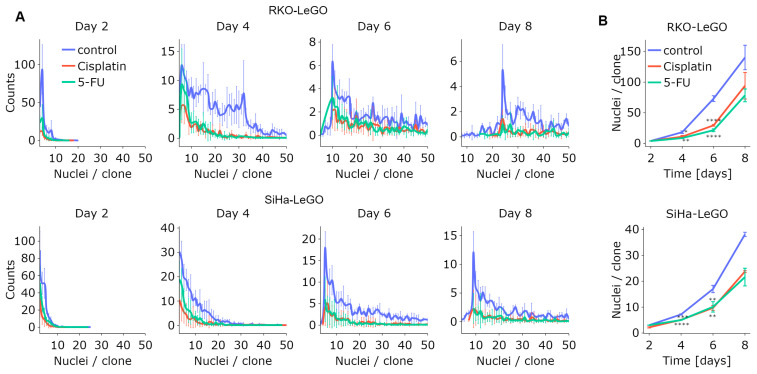
Tracking changes in number of cells per clone over time under treatment. (**A**) Changes in the distribution of clone sizes over time after treatment with 5-FU or cisplatin in RKO (top) or SiHa (bottom) cells. (**B**) Changes in the median number of cells per clone over time. N = 3. *: *p* < 0.05, **: *p* < 0.01, ***: *p* < 0.001, ****: *p* < 0.0001.

One of the key advantages of LeGO-CSA is that it eliminates the need for the staining or fixation of cells, such that the same cell cultures can be imaged at different time points, allowing for longitudinal analysis. As depicted in Figure 6A, the number of nuclei per clone increased over time for both cell types, as demonstrated by the rightward shift of the distribution peak. However, while the untreated groups exhibited a higher median number of nuclei per clone (Figure 6B), this continued to increase over time in treated samples as well. This underscores the findings obtained via the high-resolution analysis of classical CSA: while treatments may decrease the number of sufficiently large clones at a particular time point, resulting in an apparent decrease in clonogenicity using classical clone size thresholds of 50 cells, the smaller clones remain clonogenic and will likely contribute to cancer (re)growth in vivo.

**Figure 7:**
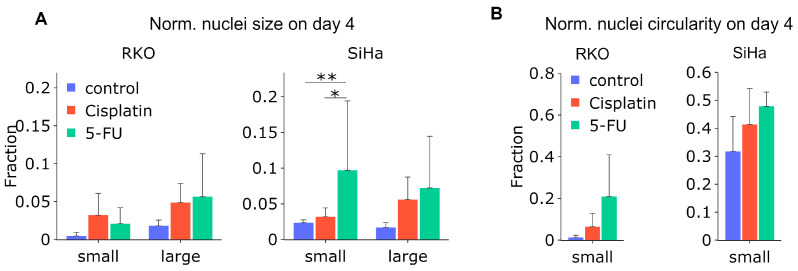
Treatment with 5-FU or cisplatin produces abnormal cell nuclei. (**A**) Fraction of abnormally small (normalized size 0–0.5) and large (2–4) nuclei after the indicated treatments; (**B**) fraction of a-circular nuclei (normalized circularity <0.9). N = 3. *: *p* < 0.05, **: *p* < 0.01.

Next, we attempted to quantify the changes in circularity and size of fluorescently barcoded nuclei over time. As the expression and intensity of the fluorescent LeGO markers are not directly correlated to DNA content, we could not determine the total DNA. Nevertheless, similarly to our strategy applied after imaging of Hoechst-stained nuclei after X-ray exposure (Figure 4A), we quantified the median nucleus size and circularity over time (Supplementary Figure S4), as well as the frequency of a-circular, abnormally small and abnormally large nuclei after treatment with 5-FU and cisplatin on day 4. It should be noted that circularity is not equivalent to roundness, which we measured in the experiments above. As depicted in Figure 7A, a large fraction of the nuclei were enlarged after 5-FU treatment in both cell types. Moreover, the frequency of small nuclei increased significantly after either cisplatin or 5-FU treatment for SiHa cells. When considering the nuclei circularity (Figure 7B), no significant difference was observed, however, even though there was a clear increasing trend of nuclei with lowered (<0.9) circularity in both cell lines.

In conclusion, these results demonstrate the utility of the fluorescent cell barcoding LeGO system, overcoming some of the challenges of traditional clonogenic assays, allowing for effective clone separation and long-term tracking, and offering more comprehensive insights into cell behavior and the efficacy of cancer treatments.

### 3.6. High-Throughput Drug Screening Using LeGO-CSA: A Proof of Concept

To evaluate the potential of LeGO-CSA in a 384-well format suitable for high-throughput drug testing, we performed a proof-of-concept screen using 20 inhibitors of kinases operating in distinct cellular pathways (Supplementary Table S1), at two concentrations (1 and 2 μm), in RKO and SiHa cells. Informed by the results presented above (Figure 5B), we imaged cells four days after seeding and treatment. In addition to assessing the clonogenic fraction, we determined the nuclei number per clone, nuclei size and circularity. We also calculated the interquartile range (IQR) of the distribution of the nuclei number per clone, which provides information about the level of clone heterogeneity, with a high IQR score representing large heterogeneity in the number of nuclei per clone. As illustrated in Supplementary Figure S5, treatments at 2 μM generally resulted in decreased clonogenicity, as well as larger changes in other nuclei parameters, except for nuclei circularity, which remained mostly unaltered under all treatments.

**Figure 8:**
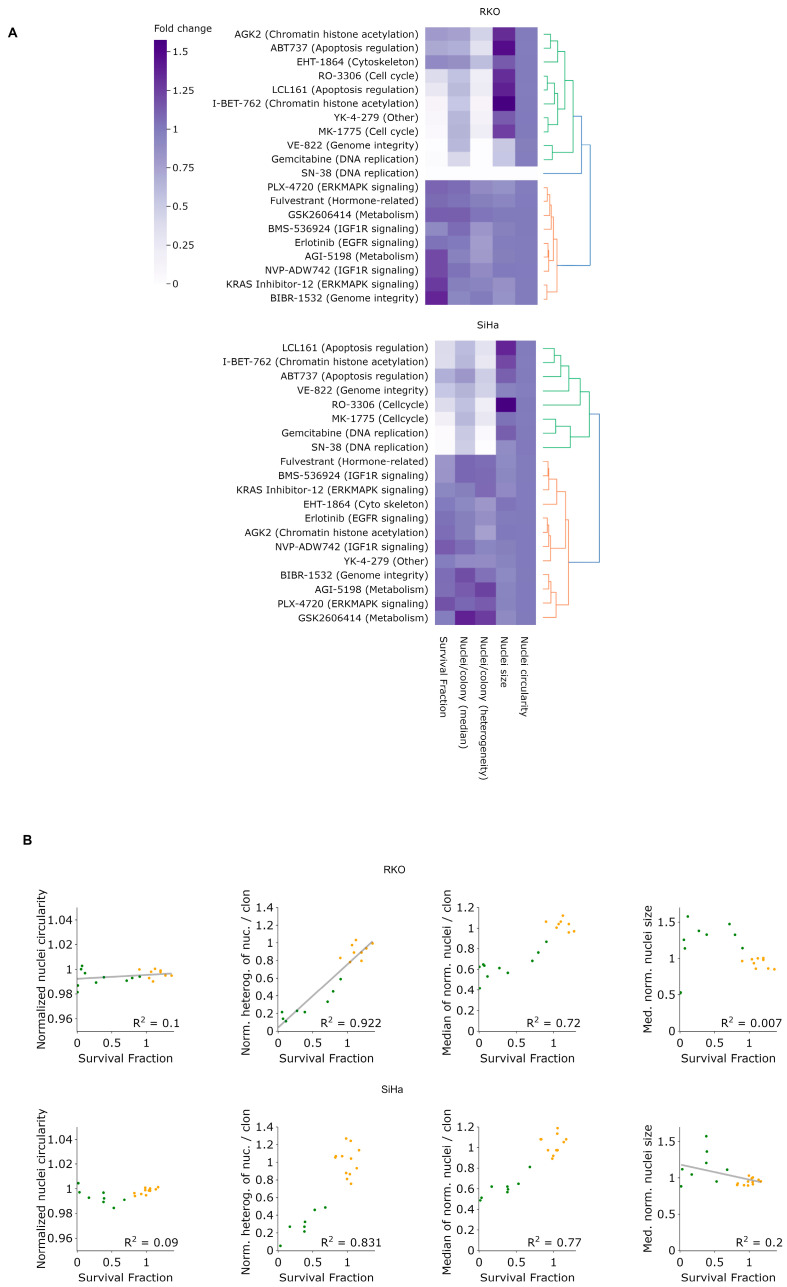
A proof-of-concept, small-scale screen using LeGO-CSA. (**A**) Heatmap representation of key parameters under treatment by 20 different drugs and the resulting respective survival fractions. Parameters include the normalized median number of nuclei per clone, normalized heterogeneity, normalized nucleus size and normalized nucleus circularity. Results were subjected to unsupervised hierarchical clustering for analysis and visualization. N = 3. (**B**) Correlation analysis between clone/nucleus characteristics and survival fraction. Various parameters of cell nuclei have been correlated with survival fraction for RKO and SiHa cells. The different drugs are clustered into groups with colors shown in clustering dendrograms of Figure 8. Different colors indicates different groups after hieratical clustering analysis. These colors are consistent with colors used in Figure 8A. N = 3.

Even though most drugs were similarly toxic to both cell lines, some noteworthy differences are observable between RKO and SiHa upon closer examination (Figure 8A and Supplementary Figure S5). For example, at the concentration of 2 μM, YK-4-279 and VE-82 lead to the almost complete eradication of RKO cells but do not affect SiHa cells.

Additionally, a positive correlation between the survival fraction and clone heterogeneity, as measured by the interquartile range (IQR), is evident under treatment by several inhibitors, such as YK-4-279, VE-822, I-BET-762 or gemcitabine, but this is likely because these treatments also considerably reduce the clone size. Notably, certain inhibitors, such as AGK2 and ABT737, demonstrate relatively low killing efficacy but significantly reduce clone heterogeneity. Such inhibitors warrant further investigation due to their potential in reducing heterogeneity, which may be translated to more effective cancer treatments. In contrast, the normalized median nucleus size and circularity did not clearly correlate with the survival fraction (Figure 8B). Intriguingly, hierarchical clustering revealed that most drugs affecting the cell cycle, chromatin, DNA replication and integrity as well as apoptosis clustered together in both cell lines, considerably affecting the nuclei size, which suggests that LeGO-CSA could yield novel insights into drug mechanisms of action in high-throughput screening contexts.

In conclusion, the LeGO-CSA approach combining CSA with fluorescence barcoding, advanced analysis methods utilizing machine/deep learning and a multi-well plate setup appears suitable for conducting large-scale high-content drug screens.

## 4. Discussion

Clonogenic survival assays, key accessories for assessing the survival and proliferative potential of cells in cancer research, face significant limitations such as their labor intensiveness, their inability for high-throughput analysis and the risk of underestimating clonogenic survival in high-density cultures. Additionally, these traditional assays often provide a binary outcome (cell survival or death), offering limited insights into nuanced cellular behavior post-treatment. Despite these challenges, CSAs remain vital in quantifying the cytotoxic effects of anticancer treatments and are integral to preclinical drug development and efficacy studies. Recognizing the limitations, this study ultimately combines the fluorescent barcoding via the LeGO system with automated microscopy and advanced image analysis to improve the accuracy, throughput, scope and content of clonogenic survival analysis, paving the way for enhanced utility in cancer research.

Pursuing our aim to increase the content and throughput of CSAs, we initially set out by applying high-resolution imaging to the classical CSA format after ionizing radiation exposure and found that it is feasible to use simple DNA staining to obtain detailed information about clonally growing cells. Our custom-developed CloneFinder 5.0 Matlab application accurately detected and quantified clonogenic survival, equating or surpassing the capabilities of manual cell counting, especially at higher radiation doses. The CloneFinder 5.0 excels in detecting smaller clones, arguably leading to a more accurate depiction of clonogenic survival.

Further, CloneFinder 5.0 offers insights into more nuanced aspects of clonogenic growth by determining additional parameters such as the cell number per clone, clone area and clone compactness. This high-content analysis not only adds depth to our understanding of cell behavior and fate post-irradiation but also highlights potential intracellular variations within the same cell line. For instance, the variations in clone compactness in the HeLa cells may point toward inherent heterogeneity in cell mobility or cell–cell or cell–surface adhesion. We also observed a shift in the relative abundance of small, low-intensity objects following radiation exposure, likely indicating an increase in small or fragmented nuclei, DNA fragments and micronuclei. These changes potentially reflect unrepaired DNA damage [23] or a high frequency of cell cycle arrest post-radiation, indicating the onset or progression of cellular senescence or apoptosis, similarly to what is measured by the often-used flow-cytometry-based “Nicoletti” assay [24]. It should be noted that this increase may overrepresent the number of actually fragmented nuclei, as a single fragmented or damaged nucleus may produce multiple fragments. It is also not possible to exclude DNA or nuclei fragments originating from dead or dying cells. The observation of a relative increase in abnormally large and misshapen nuclei with increased radiation doses underscores this hypothesis [25].

This extension of classical CSA, however, fails to address its labor intensiveness and low throughput; on the contrary, whole-plate fluorescence imaging and data processing adds a considerable amount of time, with a single plate scan taking ~6 h using our standard microscopy setup. While this could be improved using state-of-the-art, high-speed imagers, the 6-well format prohibits any high-throughput applications. Circumventing this limitation, the LeGO system opens the possibility for high-throughput clonogenic analysis in multi-well plates by leveraging distinct color barcoding for individual cell clones. This system effectively overcomes the challenge of distinguishing overlapping clones in high-density cultures, a problem often encountered in classical CSA, as well as in screening well plates. By employing this system, we managed to standardize the optimal seeding density and conduct longitudinal analysis, allowing us to trace the evolution of clones over time without necessitating cell fixation or staining. Notably, we were able to validate the assay in a proof-of-concept small-scale screen using the screening-friendly 384-well plates, and, to our knowledge, this is the first technique that allows this, as previously developed high-throughput CSAs all employed 96-well plates [26,27,28,29,30,31]. Because of the limited scope, in these experiments, we decided to acquire z-stack images which were preprocessed using custom software to pick the in-focus image slice for further analysis. This produced high-quality images, albeit at a cost of time—a full-plate scan took ~6 h, with preprocessing taking a further hour per plate. A comparable single-image-plain, full-plate scan requires only ~30 min using our Leica Thunder microscope and could be further considerably reduced (to few minutes per plate) using state-of-the-art spinning disk high-throughput imagers, rendering our approach compatible with the high-throughput and high-content analysis of large drug libraries. While this proof-of-concept study used a limited panel of inhibitors, it thus outlines a promising path toward large-scale drug efficacy studies, wherein the clonogenic survival potential of cells can be assessed rapidly and comprehensively, in parallel with other parameters of key relevance in cancer.

Further improvements to LeGO-based CSA could be contemplated. For instance, the analysis yielded a lower survival fraction compared to traditional CSA in drug-treated samples, likely due to the different cell adherence conditions in plastic and glass-bottomed plates. This difference suggests a need for more comprehensive optimization of culture conditions, especially related to cell-culture vessels, in LeGO-based analysis. Additionally, the application of our methodology to additional cell lines would help assess its generalizability and broaden its scope for various applications in cellular and cancer biology. On the other hand, even though we studied different cell lines in isolation here, the barcoding strategy could be repurposed to study cell cultures of different compositions, e.g., containing (multiple types of) non-transformed as well as cancer lines of a different genetic makeup. The assay could also potentially benefit from increasing the number of distinct barcoded subpopulations. One possible approach would be to use “rainbow” cell cultures, wherein each transduced cell has a potentially unique color, resulting from different levels of expression of each of the three fluorescent reporters, as in the original set of experiments describing the LeGO system [15,21]. This would have an additional benefit of shortening the time required for generating stable cell lines, as generating “pure” single-color populations would not be required. Additionally, this approach could eliminate, or at least significantly reduce, any side-effects of selecting and sorting predefined color sub-populations.

## 5. Conclusions

In summary, our study introduces two novel, complementary approaches to enhance the resolution and scale of clonogenic assays. The adaptation and further refinement of these methods can open new avenues for exploring cellular behaviors, understanding clonal dynamics and predicting treatment responses in the context of cancer biology.

## Data Availability

All data, visualizations and figures generated during this study are accessible via the FiglinQ data lifecycle platform. They can be accessed using the following link: https://create.figlinq.com/~h.qian/378/ (accessed on 25 September 2023).

## Supplementary Materials

The following supporting information can be downloaded at: https://www.mdpi.com/article/10.3390/cancers15194772/s1, Supplementary Figure S1: Absolute counts of (A) clone compactness, (B) nuclei number per clone and (C) normalized clone size for Du145, Hela, RKO and SiHa cell lines exposed to 0, 2 and 6 Gy X-ray irradiation. N = 3; Supplementary Figure S2: Examples of micronuclei detected in CloneFinder 5.0 through precise segmentation; Supplementary Figure S3: Absolute counts of (A) the normalized nucleus size and (B) nucleus roundness for Du145, Hela, RKO and SiHa cell lines exposed to 0, 2 and 6 Gy X-ray irradiation. N = 3; Supplementary Figure S4: Changes in the normalized nucleus circularity and size over time under different treatments for (A) RKO and (B) SiHa cell lines. N = 3; Supplementary Figure S5: Full results of a limited compound screen using RKO and SiHa cells treated with 20 different kinase inhibitors at concentrations of 1 and 2 μM. Quantified characteristics include survival fraction, nuclei number per clone, the heterogeneity of clones, nucleus size, nucleus circularity and nucleus maximum and minimum diameter. N = 3; Supplementary Table S1: Kinase inhibitors; Supplementary File S1: Qupath image analysis.
