# Supplementary material for: High-Content and High-Throughput Clonogenic Survival Assay Using Fluorescence Barcoding
Source: Cancers (Basel). 2023 Sep 28;15(19):4772. doi: 10.3390/cancers15194772 (PMC10571559; doi:10.3390/cancers15194772)
Supplement: Supplementary file 1 [file cancers-15-04772-s001.zip › File S1. Qupath image analysis.pdf]

The image analysis process was undertaken using the open-source software, QuPath, accessible at https://qupath.github.io/. Here's a breakdown of the procedure we used :

1. **Initialization:** We first created a project in QuPath by loading the images into an empty directory.
2. **Nuclei Detection using Stardist:** Using QuPath's built-in deep learning algorithm, Stardist, we selected the well area on the plate to initiate automated detection of structures resembling nuclei.

a.

```
import qupath.ext.stardist.StarDist2D
def server = getCurrentServer()

import qupath.lib.gui.QuPathGUI
def projectpath = QuPathGUI.getInstance().getProject().getBaseDirectory()

// Specify the model file (you maybe need to change this!)
var pathModel = projectpath.toString()+'/models/dsb2018_heavy_augment.pb'

def cal = server.getPixelCalibration()

var downscale=1
var threshold=0.3             // Probability (detection) threshold
var cellexpansion=0           // Approximate cells based upon nucleus expansion (0=off)

try {
    var stardist = StarDist2D.builder(pathModel)
            .preprocess (
                ImageOps.Channels.mean()
            )
            .threshold(threshold)          // Probability (detection) threshold
            //.channels(0)             // Select detection channel
            .normalizePercentiles(1, 99)   // Percentile normalization
            .pixelSize(cal.getAveragedPixelSizeMicrons()*downscale) // Resolution for detection
            .cellExpansion(cellexpansion)  // Approximate cells based upon nucleus expansion
            .cellConstrainScale(1.5)       // Constrain cell expansion using nucleus size
            .measureShape()                // Add shape measurements
            .measureIntensity()            // Add cell measurements (in all compartments)
            .includeProbability(true)      // Add probability as a measurement (enables later filtering)
            .doLog()                       // Use this to log a bit more information while running the script
            //.tileSize(1024)                // Specify width & height of the tile used for prediction
            //.ignoreCellOverlaps(false)     // Set to true if you don't care if cells expand into one another
            //.nThreads(4)                   // Limit the number of threads used for (possibly parallel) processing
            //.simplify(1)                   // Control how polygons are 'simplified' to remove unnecessary vertices
            //.createAnnotations()           // Generate annotation objects using StarDist, rather than detection objects
            //.constrainToParent(false)      // Prevent nuclei/cells expanding beyond any parent annotations (default is true)
            .classify("Nuclei")            // Automatically assign all created objects as 'Tumor'
            .build()

    // Run detection for the selected objects


    clearSelectedObjects(true);
    clearSelectedObjects();
    resetSelection();
    createAnnotationsFromPixelClassifier("Well", 10000.0, 0.0, "DELETE_EXISTING", "SELECT_NEW")

    var imageData = getCurrentImageData()
    var pathObjects = getSelectedObjects()
    if (pathObjects.isEmpty()) {
        Dialogs.showErrorMessage("StarDist", "Please select a parent object!")
        return
    }
    stardist.detectObjects(imageData, pathObjects)

```

b. 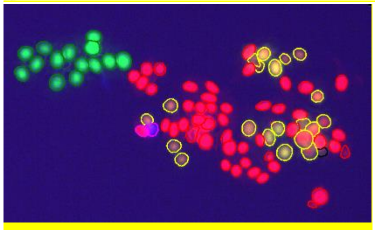 nuclei was nicely detected after running this script

c. 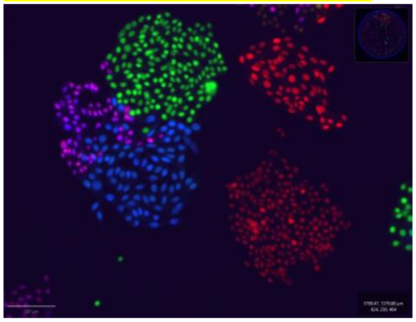 raw microscopy images

d. 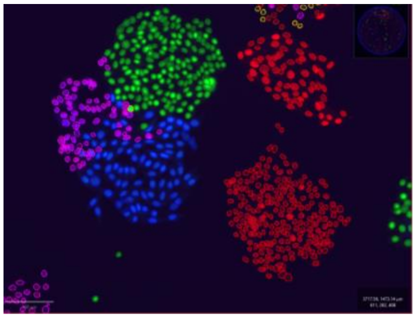 nuclei detection

e. 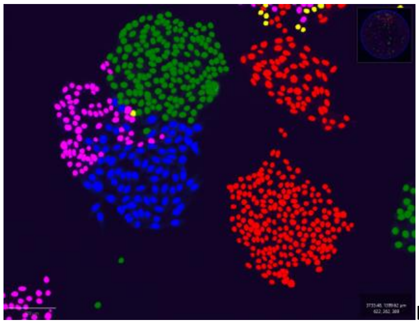nuclei segmentation with mask

3. **Manual Annotation for Nuclei Classification:** While Stardist helped in identifying potential nuclei, it did not distinguish between them based on color. Researchers then manually annotated each nucleus by creating objects in QuPath. The detected nuclei were grouped into different colored object groups. Any incorrectly detected elements, such as dirt or debris, were placed in a 'trash' group.

   a. 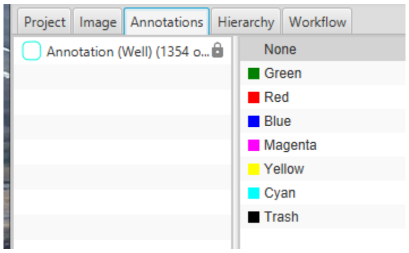

4. **Training the RandomForest Model:** We utilized the manually annotated color groups to train a RandomForest model. This model considered various parameters, such as RGB channel fluorescence intensity, nucleus size, nucleus circularity, and coordinates. It's worth noting that the model's training was a collaborative effort involving multiple researchers, and upon completion, it was saved for future applications. While other machine learning and deep learning models were explored, our evaluations found the RandomForest to be the most effective.
5. **Application on All Images:** After training, the RandomForest model was then applied to all the images for nuclei detection.

Codes in Qupath:

```
import qupath.ext.stardist.StarDist2D

def server = getCurrentServer()

import qupath.lib.gui.QuPathGUI

def projectpath = QuPathGUI.getInstance().getProject().getBaseDirectory()

// Specify the model file (you maybe need to change this!)

var pathModel = projectpath.toString()+'/models/dsb2018_heavy_augment.pb'


def cal = server.getPixelCalibration()
```

```
var downscale=1
var threshold=0.5          // Probability (detection) threshold
var cellexpansion=0        // Approximate cells based upon nucleus expansion (0=off)

try {
   var stardist = StarDist2D.builder(pathModel)
          .preprocess (
             ImageOps.Channels.maximum()
          )
          .threshold(threshold)        // Probability (detection) threshold
          //.channels(0)           // Select detection channel
          .normalizePercentiles(1, 99)   // Percentile normalization
          .pixelSize(cal.getAveragedPixelSizeMicrons()*downscale) // Resolution for detection
          .cellExpansion(cellexpansion)  // Approximate cells based upon nucleus expansion
          .cellConstrainScale(1.5)      // Constrain cell expansion using nucleus size
          .measureShape()              // Add shape measurements
          .measureIntensity()          // Add cell measurements (in all compartments)
          .includeProbability(true)     // Add probability as a measurement (enables later filtering)
          .doLog()                     // Use this to log a bit more information while running the script
          //.tileSize(1024)              // Specify width & height of the tile used for prediction
          //.ignoreCellOverlaps(false)    // Set to true if you don't care if cells expand into one another
          //.nThreads(4)                 // Limit the number of threads used for (possibly parallel) processing
          //.simplify(1)                 // Control how polygons are 'simplified' to remove unnecessary vertices
          //.createAnnotations()         // Generate annotation objects using StarDist, rather than detection
objects
          //.constrainToParent(false)     // Prevent nuclei/cells expanding beyond any parent annotations
(default is true)
          .classify("Nuclei")          // Automatically assign all created objects as 'Tumor'
```

```
        .build()

    // Run detection for the selected objects



    clearSelectedObjects(true);
    clearSelectedObjects();
    resetSelection();
    createAnnotationsFromPixelClassifier("Well", 10000.0, 0.0, "DELETE_EXISTING", "SELECT_NEW")

    var imageData = getCurrentImageData()
    var pathObjects = getSelectedObjects()
    if (pathObjects.isEmpty()) {
      Dialogs.showErrorMessage("StarDist", "Please select a parent object!")
      return
    }
    stardist.detectObjects(imageData, pathObjects)

    setImageType('FLUORESCENCE');
    runObjectClassifier("RKO_test_rt_train");

    println 'Done!'
  }
  catch(Exception e) {
    Dialogs.showErrorMessage("Stardist",e.getMessage())
    println 'Please Correct the Script!'
  }
```
