# Supplementary material for: High-Content and High-Throughput Clonogenic Survival Assay Using Fluorescence Barcoding
Source: Cancers (Basel). 2023 Sep 28;15(19):4772. doi: 10.3390/cancers15194772 (PMC10571559; doi:10.3390/cancers15194772)
Supplement: Supplementary file 1 [file cancers-15-04772-s001.zip › Supplementary Figures .pdf]

High-content and high-throughput clonogenic survival assay using fluorescence barcoding

Haibin Qian[1,3,†], Selami Baglamis[2,3,4,5,†], Fumei Redeker[1,3], Julia Raaijman[1,3], Ron Hoebe[1,3], Vivek M Sheraton[2,3,4,5,6], Louis Vermeulen[2,3,4,5], Przemek Krawczyk[1,3,*]

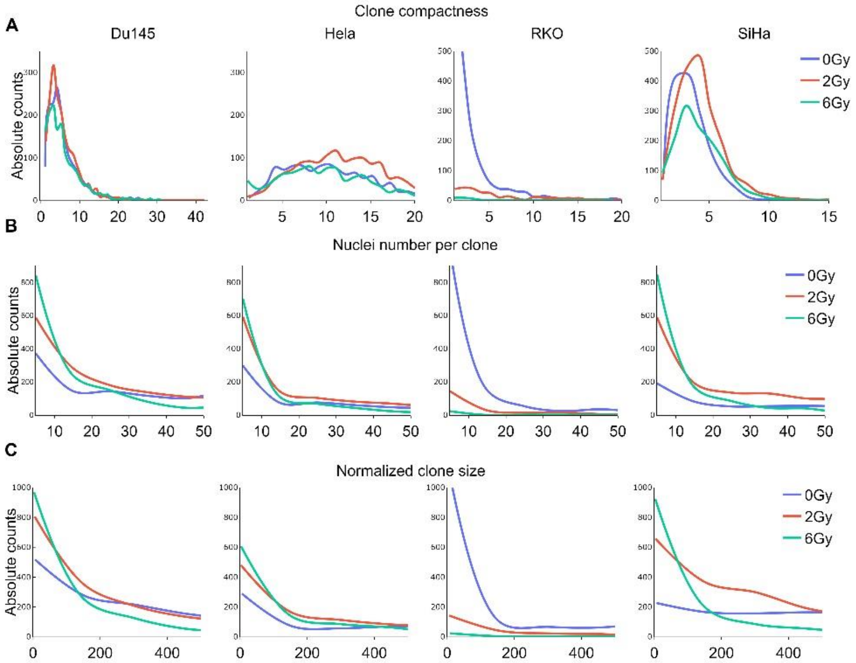

Supplementary Figure S1. Absolute counts of (**A)** clone compactness, (**B)** nuclei number per clone, and (**C)** normalized clone size for Du145, Hela, RKO, and SiHa cell lines exposed to 0, 2, and 6 Gy X-ray irradiation. N=3

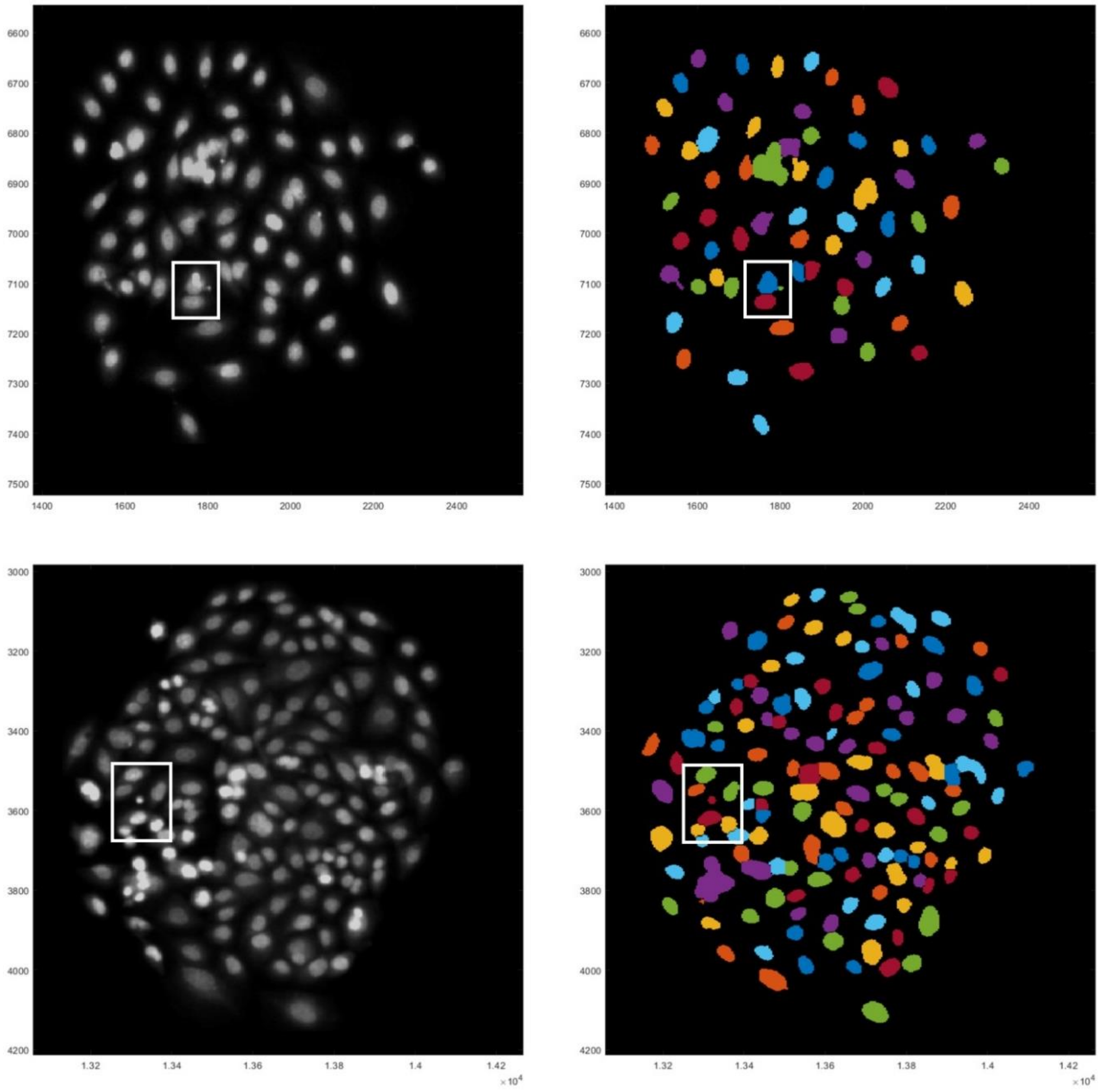

Supplementary Figure S2. Examples of micronuclei detected in CloneFinder through precise segmentation.

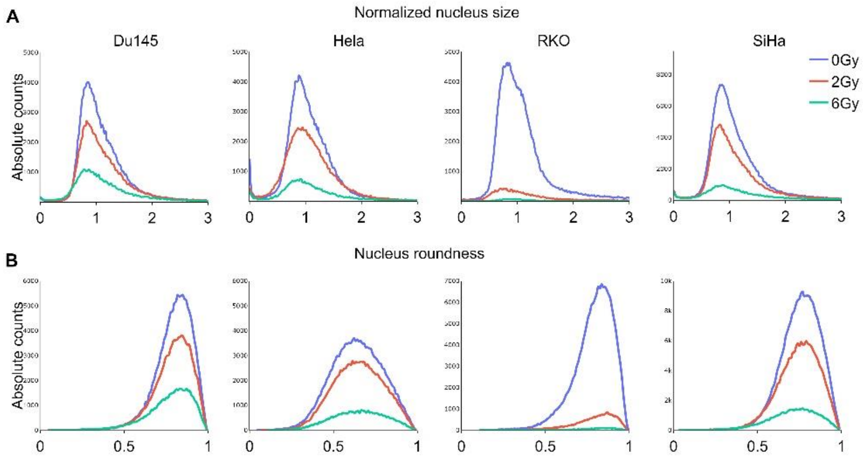

Supplementary Figure S3. Absolute counts of (**A**) the normalized nucleus size and (**B**) nucleus roundness for Du145, Hela, RKO, and SiHa cell lines exposed to 0, 2, and 6 Gy X-ray irradiation. N=3

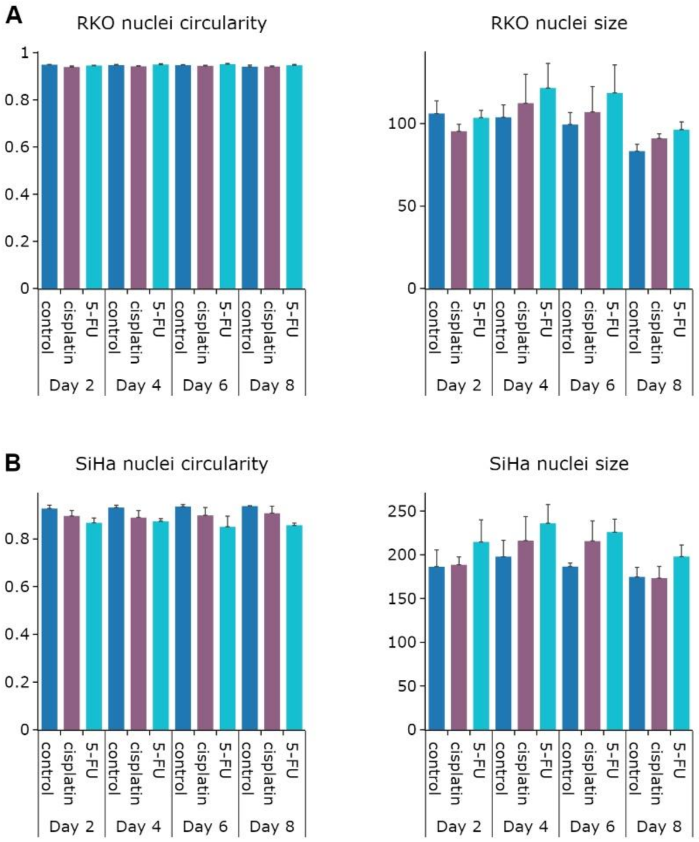

Supplementary Figure S4. Changes in the normalized nucleus circularity and size over time under different treatments for (**A**) RKO and (**B**) SiHa cell lines. N=3

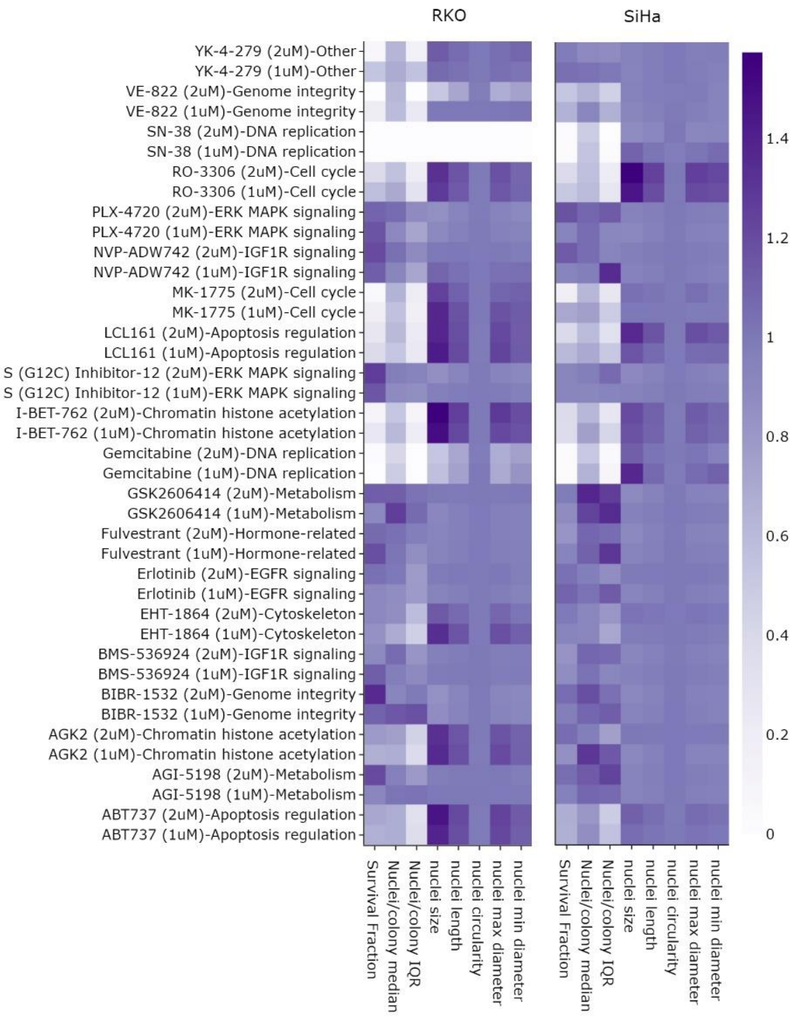

Supplementary Figure S5. Full results of a limited compound screen using RKO and SiHa cells treated with 20 different kinase inhibitors at concentrations of 1 and 2 μM. Quantified characteristics include survival fraction, nuclei number per clone, the normalized heterogeneity of clones, nucleus size, nucleus circularity, and nucleus maximum and minimum diameter. N=3
